# Play Therapy as an Intervention in Hospitalized Children: A Systematic Review

**DOI:** 10.3390/healthcare8030239

**Published:** 2020-07-29

**Authors:** María José Godino-Iáñez, María Begoña Martos-Cabrera, Nora Suleiman-Martos, José Luis Gómez-Urquiza, Keyla Vargas-Román, María José Membrive-Jiménez, Luis Albendín-García

**Affiliations:** 1 Faculty of Health Sciences, University of Granada, Avenida Ilustración 60, 18016 Granada, Spain; mariajosegdns1@gmail.com (M.J.G.-I.); jlgurquiza@ugr.es (J.L.G.-U.); 2 San Cecilio University Hospital, Andalusian Health Service, Avenida de la Ilustración s/n, 18016 Granada, Spain; begomartos90@gmail.com; 3 Faculty of Health Sciences, University of Granada, Cortadura del Valle s/n, 51001 Ceuta, Spain; 4 Faculty of Psychology, University of Granada, Campus Universitario de Cartuja s/n, 18071 Granada, Spain; keyvarom@ugr.es; 5 Ceuta University Hospital, National Institute of Health Management, Loma Colmenar s/n, 51003 Ceuta, Spain; mariajose.membrive@gmail.com; 6 Andalusian Health Care, Avenida del Sur 11, 18014 Granada, Spain; lualbgar1979@ugr.es

**Keywords:** game, hospitalization, nursing, pediatrics, therapeutic play

## Abstract

Background: Hospitalization disrupts children’s lives and can produce feelings such as anxiety, fear, or pain. Playing is an important part of children’s lives. Thus, it is necessary to ensure holistic care during the process, including play therapy. The aim of this study was to analyze the effect of therapeutic play in hospitalized children. Methods: A systematic review was performed. The search was conducted in CINAHL (Cumulative Index of Nursing and Allied Health Literature), CUIDEN, and PubMed (Medline). The search equation was “pediatric nurs* AND play therapy”. The search was performed in March 2020. Results: *n* = 14 studies were included in the review. The studies reveal that the application of therapeutic play in hospitalized children decreases postoperative pain, improves behavior and attitude, and reduces anxiety during the hospital stay. Conclusions: play therapy has a beneficial impact on the care of hospitalized children and should be implemented in pediatric units after assessing the resources and training needed for pediatric nurses.

## 1. Introduction

In the European Letter for Hospitalized Children approved in 1986, several rights were adopted, highlighting the “right of the child to receive information adapted to his age, mental development, emotional and psychological state” and “the right to dispose of age-appropriate toys, books, and audiovisual media during his stay in the hospital” [1].

Childhood is a key stage in human life that influences the development of the person. In some cases, this phase may be interrupted by adverse events such as diseases, pathologies, painful and invasive procedures, trauma, or prolonged hospitalizations [2].

When hospitalization is necessary, the child may perceive this situation as traumatic and it can alter their emotional development, as they are separated from their daily and family environment to face an unknown process with painful interventions and restrictions. During the hospitalization, the child may experience negative behaviors and emotions, such as stress, fear, anxiety, pain, insecurity, and uncertainty [3,4].

Furthermore, if the cause of hospitalization is a cancer diagnosis, these issues may be more complex, as it is a complex disease that can be accompanied by prolonged treatments with intense side effects and, probably, long and frequent hospital stays [5,6].

In the hospitalization period, play therapy is essential, not only because children like to play, but also because it facilitates interventions by health professionals [7]. Play is an essential activity in a child’s life, so, in the context of hospitalization, it can help children to face this unknown situation, express their emotions and concerns, feel more comfortable and safer, become familiar with medical techniques, and make decisions [6]. It also helps communication and promotes the development and recovery of the child’s own individuality [8].

Play therapy can be defined as the set of interventions to promote children’s wellbeing during the hospitalization or the play activities structured depending on the child’s health condition, age, and development [6]. Therefore, it is a planned activity with a purpose and not just a recreational activity [7].

Pediatric nurses can use play as a care strategy for hospitalized children. It is very important for nurses to know and use play in children’s care, as it can have numerous advantages in hospitalization. The function of catharsis, which means the relief of anxiety and which is the basis for play therapy, can be highlighted [9]. Play activities performed by health professionals can also improve the nurse–child relationship, increasing confidence during the hospitalization period [8].

Therefore, it is important to know the effectiveness of play therapy to transform children hospitalization units into a pleasant, attractive, and playful environment in order to achieve better results and the humanization of care [10].

The aim of the study was to assess the impact and effectiveness of play therapy on the care of hospitalized children. The PICO question that guided this review was what are the effects of play therapy for hospitalized children?

## 2. Materials and Methods

A systematic review was carried out following the PRISMA (Preferred Reporting Items for Systematic Reviews and Meta-Analyses) guidelines.

Information sources and search equation: For the search, the CINAHL (Cumulative Index of Nursing and Allied Health Literature), CUIDEN, and PubMed (Medline) databases were consulted, restricting the results to documents published in the last 10 years. The search was performed in March 2020, and the search equation was “pediatric nurs* AND play therapy”. The descriptors of the search equation were obtained from the thesaurus Medical Subject Headings (MeSH).

Inclusion and exclusion criteria: Primary quantitative studies evaluating the effects of play therapy on hospitalized children published in English, Spanish, or Portuguese in the last 10 years were included. Studies that were not related to the topic, that had adult samples, and those in which the opinion of parents or nurses was studied were excluded.

Selection process, critical reading, and level of evidence: The selection of the studies was conducted by two researchers independently in four stages. First, the title and abstract of the studies were read. The second stage was reading the full text. Then, the documents were critically read in order to assess and identify biases in the methodology. Finally, a reverse search was performed from the previously chosen articles. A third member of the team was consulted in case of disagreement. The Oxford Centre for Evidence Based Medicine (OCEBM) classification was used to define the level of evidence and the degree of recommendation for each study.

Study variables and data analysis: The following variables were collected from each study: year of publication, country where the study was carried out, study design, sample size, play therapy intervention, and main results. A descriptive analysis of the studies was performed.

## 3. Results

**Figure 1:**
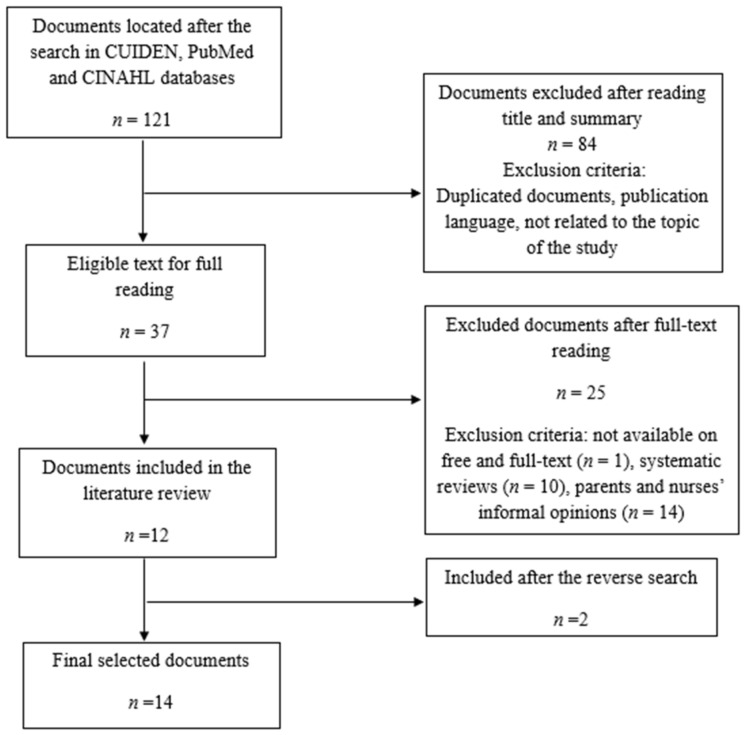
Study selection diagram.

The search showed 121 documents, and 84 articles were excluded after reading the title and abstract because they were duplicated documents or they had no relation to the study topic. After this phase, 37 full-text publications remained, of which 25 were excluded as they did not meet the inclusion criteria. Therefore, 12 studies were included in the review. After the reverse search in the previously selected documents, two more articles were added. Finally, the sample consisted of *n* = 14 studies. The selection process is detailed in Figure 1.

The total population in the selected studies was *n* = 856 children. Of the included studies, six were clinical trials, one was quasi-experimental, and seven were descriptive studies.

A total of 50% of the studies included in this review were conducted in South America. Regarding the year of publication, 28.6% were published in 2016. The larger sample size was *n* = 165 children, and the smallest was *n* = 5 children. Regarding the sex of the sample, the male gender predominates. All of interventions with play therapy were added to the usual care routine as an extra intervention. Table 1 summarizes the characteristics of the included studies.

### 3.1. Therapeutic Play in Surgically Operated Children

Some studies have shown that therapeutic play, in different modalities, produces a decrease in pain in children after surgery [11,12,13,14]. When comparing the level of pain between boys and girls, the scores were higher in boys and in the youngest (1–3 years) [12]. According to the study by Yayan et al. [11], the level of pain is higher in those children whose parents have a high level of anxiety.

Regarding the behavior of children in the postoperative period, the study by Kiche and Almeida [13] shows that after therapeutic play the behaviors manifested by children are those of greater adaptation and acceptance to the procedures (change of postoperative dressing). Behaviors such as “smile”, “play”, “relaxed posture”, or “help the professional spontaneously” increased.

Regarding anxiety and negative emotions, two studies [14,15] reported that therapeutic play, applied before surgery, decreased the levels of these feelings on the surgery day.

The duration of the play therapy sessions for children who were operated on mainly had a duration of around 1 h and were done before [14,15] the surgery and after [11,12,13] the surgery.

### 3.2. Play Therapy in the Care of Children with Acute or Chronic Pathologies

Other studies have pointed out that therapeutic play sessions in hospitalized children can improve their behaviors, as this distracts and amuses them during their hospital stay [16,17,18,19]. In addition, the study by Al-Yateem et al. [20] informs that play therapy decreases the level of anxiety in boys during their hospital stay.

Regarding the behavior of children in specific nursing techniques, such as the puncture or administration of intravenous medication, some studies [18,19] indicate the importance of applying play therapy. After the sessions, the children were more calm and relaxed, cooperating with the professional who performed the technique. It also improved the level of trust in the nurse–child relationship [16,17,18].

Play therapy helps children express and communicate their feelings and emotions through toys. It also makes them understand the process and the need for hospitalization [17].

The duration of the play therapy sessions was not indicated in all the studies [16,17,19], and it was 30 min sessions in the studies that included this information [18,20]

### 3.3. Play Therapy in Children Diagnosed with Cancer

Some studies inform that cancer produces feelings of fear, anxiety, and insecurity for the future and depressive symptoms in children [21,22]. Through play interventions, it has been found that depressive symptoms can be reduced in children with cancer who are hospitalized for long periods [21]. In addition, it helps to express and diminish the feelings mentioned above [22].

Other studies in children receiving outpatient chemotherapy showed that play therapy improved children’s attitude to the disease [23,24]. The study by Artilheiro et al. [23] showed that, after the play sessions, children had positive behavior (relaxed posture and smile) and decreased behaviors that indicated fear, anxiety, and anger (tension, crying, screaming, beating). Furthermore, children became involved and collaborative in the processes of their disease, increasing their trust in the professionals [24].

One study had 30 min play sessions 5 days a week [21], other had sessions from 1 to 3 h [24], and others did not inform about the duration of the session [22,23].

## 4. Discussion

The aim of the study was to understand the impact of play therapy on hospitalized children. The results of the studies indicated that it has multiple benefits, such as reducing anxiety and pain, children having less negative emotions, better children–health professional relations, or improving children’s collaboration in their treatments. This review includes information about the use of play therapy for different children’s pathologies or hospitalization reasons and describes different kinds of interventions. It also suggests what the characteristics of play therapy interventions based on the reviewed literature should be and what are the main future research lines that have not been addressed yet.

Play therapy theoretical models indicate that its effectiveness is based on six points: the therapeutic relationship, the diagnostic opportunities, breaking down defense mechanisms, facilitating articulation, therapeutic release, and anticipatory preparation [25]. Furthermore, the processes that can be changed or affected by play therapy belong to three domains: cognitive, affective, and interpersonal [25]. The cognitive domain refers to the awareness and domination of beliefs and ideas which can be changed with processes such as skill development, schema transformations, and symbolic exchanges; the affective domain, related to emotion regulation, uses processes such as the abreaction of affective education; the interpersonal domain, related to relations and support, can include processes such as support and validation and the corrective relationship with the therapist [25]. The professionals, knowing which domain of the children they want to improve or change, can focus their play therapy intervention on the application of those processes inside the games. Based on the literature review, some characteristics that are common in almost all of the studies [11,12,13,14,15,16,17,18,19,20,21,22,23,24] and that should be taken into account in a model for play therapy interventions with children at the hospital are the following: the duration should be between 30 min and 1 and half hour; dolls/toys should be used for symbolic play (the dolls/toys receive the treatment/interventions that are going to be performed on the child); the child should repeat the procedures with the dolls/toys after seeing the professional doing it; sanitary material should be included in the sessions; the child should have the opportunity to ask questions and decline to play if they want to. Based on these basic standards, the session should be creatively adapted to what the healthcare staff want to achieve.

Hospitalization periods during childhood can affect children’s behavior, as they are subjected to unknown procedures without understanding the need for these treatments. Numerous studies show that play therapy helps to improve these behaviors; this may be due to the fact that, through play, children can understand and accept the situation they are living in [26,27,28] and learn to express feelings and emotions about the hospitalization procedures [28,29,30]. Additionally, the play interventions distract and amuse the children, making them forget where they are and transporting them to a world of illusion and happiness [26,27,28,29,30].

The diagnosis and treatment of cancer in children involves long periods of hospital stay that can lead to an alteration in their development and psychosocial status, so it is necessary to provide holistic care that is not only focused on the oncological process. Several studies show that, with play therapy, children develop a trust relationship with nurses, allowing nurses to know the needs of the patient during the process [31]. In addition, children develop coping strategies, express their fears and concerns through play, and gain knowledge about their illness and treatment [31,32].

Other studies show that therapeutic games decreased anxiety in children who experienced the game session in the hospital environment. After the play sessions, children feel safer because they learn the environment and the procedures [26,27,30,33,34]. In some sessions, nurse techniques are simulated in the toys, which allowed the child to move from a passive to an active role, reducing the fear of these procedures [27,29] and establishing a positive child–nurse relationship [26,29,30].

Similar positive results have been obtained with play therapy in adults and elderly people, although literature that studies the impact of ludic activities in adults with pathology is scarce. Despite this, some studies [35,36,37,38] show that recreational interventions are beneficial for treatment in adults, indicating that creative play and psychological and physical therapies (aromatherapy, hypnosis, physical activity...) performed in adults with cancer can relieve stress, anxiety, and pain intensity; achieve a greater adherence to treatment; promote positive coping skills; and improve the quality of life [35,36]. In addition, the Bellin Health Cancer Center has developed a program called “Bellin Expressive Arts in Medicine” that consists of providing patients with comprehensive care [38], encouraging creative activities in order to promote a healing environment, and increasing the hope of these patients [39]. Additionally, other researches show the effectiveness of playing activities in adults; the study by Mimi et al. [36] shows an improvement in patients with chronic pain, and the study by Saywell et al. [37] points out the effect of recreational interventions in the rehabilitation of adults with acquired brain injury, improving the balance and independence of these patients. On the other hand, some studies show the benefits of doll therapy in elderly people with dementia [40,41]. This therapy decreases negative behaviors (anxiety, agitation...), increases positive behaviors and happiness, and improves the well-being of these people.

It is important to take into account that the effectiveness and effects of the play therapy can vary depending on the professional’s competencies and ability to engage and the children’s development level [25].

This study has some limitations. First, half of the studies included in this review are observational studies that have a lower level of evidence. Finally, we could not do a meta-analysis because the study variables and the way that they were measured were different between the studies.

The research indicates a trend of using play therapy mainly in children who are in the perioperative period and in children with cancer. Most of the games are based on showing the children different procedures that they will receive during their hospitalization [5,6,7,8,9,10,11,21,22,23,24]. Some major problems that should be covered it is the influence of play therapy for chronic and psychological pathologies’ acceptance and management in children and not only for hospital procedures. It would also be interesting to develop studies to verify whether the pediatric units have the necessary resources to carry out the play intervention, and studies to verify the knowledge of pediatric nurses regarding therapeutic play. Additionally, cost-effectiveness studies would be of interest.

The most important implications for the practice of this study is to publicize the beneficial effects of play therapy on hospitalization during childhood and the main characteristics that play therapy should have, as indicated above. The results of this review will make parents and nurses see the importance of playing to improve the well-being of children, despite illness.

In addition, this study will raise awareness of the importance of developing a relationship of trust between the nurse and the child through play, avoiding health professionals being seen as enemies by children and becoming people with whom children can express their feelings, fears, and concerns. Finally, it is important to emphasize the importance of children participating in the disease process, because in this way they better understand and accept the situation.

## 5. Conclusions

Play therapy has shown benefits in hospitalized children, such as reducing anxiety during the hospitalization process; reducing pain; improving the relationship with health professionals; improving the behavior and attitude of children to the disease and the procedures; and relieving feelings of fear, anxiety, and insecurity. Pediatric units should train their staff in the use of play therapy in order to obtain its positive effects. Additionally, future research should analyze which play therapy/games are more effective, comparing different sessions to each other instead of comparing only the play therapy with the usual care.

## Figures and Tables

**Table 1:** Summary of the included studies (*n* = 14).

Author, Year of Publication and Country	Study Type	Sample	Play Therapy Intervention	Results	EL/RG
Al–Yateem et al., 2016 [20] United Arab Emirates	Randomized clinical trial	*n* = 165 children in the pediatric unit hospitalized for at least 3 days. Mean age: 5.24 ± 1.01. 53% girls.	2 phases in the experimental group. Phase 1: the first days of admission (3 days) children complete an anxiety survey and no change was done to their care routine. Phase 2: children play in the hospital or in their beds with different toys (balloons, coloring books, face painting, play dough, bubbles and storytelling) twice a day for 30 min. This phase started the second day.	At the end of the intervention, the anxiety scores in the intervention group were significantly lower than in the control group.	1b/A
He et al., 2015 [14] Singapore	Randomized clinical trial	*n* = 95 children undergoing surgery. Age: 9.74 years. 64.25% boys.	The children in the experimental group received an individual session (1 h) of play therapy 3–7 days before the surgery. This session consisted of watching a video using a doll to show preoperative procedures, induction to anesthesia, and return after surgery. They received a manual describing the medical objects and equipment. In addition, each child was given an O_2_ mask and a cannula to take home. All the children received regular care from health professionals prior to surgery. The play intervention was a specific intervention for the intervention group and was not included in the routine care. The data was collected 3–7 days before the surgery and 24 h post-surgery.	Children in the experimental group showed significantly fewer negative emotions prior to anesthesia induction and less postoperative pain compared to the control group. Changes in the preoperative anxiety were not significant.	1a/A
Li et al., 2011 [21] China	Randomized clinical trial	*n* = 122 children admitted to the Pediatric Oncology Unit for a period of 14 months. Mean age: 12.1 years. 52.86% boys.	The control group received the usual care (information about the cancer treatment, effects, and complications; diet...). The experimental group, in addition to usual care, played virtual reality computer games for 30 min 5 days a week. In the virtual room, they played with videos that were projected on walls, floors, or ceilings with the software Play Motion.	After 7 days using this type of game, depressive symptoms decreased in the experimental group and increased in the control group.	1b/A
Li et al., 2014 [15] Hong Kong	Randomized clinical trial	*n* = 108 children admitted for elective surgery. Age: 7 to 12 years. 67.45% boys.	All the participants received information and routine care prior to surgery. The experimental group received an extra play therapy intervention in groups of 5 children for 1 h. The session took place in the operating room, where an explanation and a simulation of the surgery procedures using a doll was performed for children and parents and then repeated by the children. The level of anxiety was evaluated 3 times: before the intervention, after surgery, and after surgery.	Children in the experimental group showed significantly lower levels of anxiety than those in the control group.	1a/A
Teksoz et al., 2016 [16] Turkey	Randomized clinical trial	*n* = 30 children in the Pediatric Unit hospitalized at least 3 days. Mean age: 10 ± 1.50 years. 50% girls.	In the experimental group, children played in a room with unused medical material for creating toys. Children were free to enter in the room and play. There was no specific duration for the intervention. In the control group, the children received nursing routine care and attended a room available in the unit where there were a computer and other toys.	After the play intervention, the levels of satisfaction of the children in the experimental group were significantly higher. In addition, the children showed a hospital stay as positive. The nurse–child relationship was improved. The mean duration of the hospitalization was 4.9 days.	1b/A
Ullan et al., 2012 [12] Spain	Randomized clinical trial	*n* = 95 children who were surgically treated. Mean age: 3.9 years. 69% were boys.	The researchers informed the parents about the importance of distracting the children after the operation and gave them a toy after the operation. The toy was a plush rabbit dressed as a doctor for two reasons: because children usually show affection towards stuffed animals, and because evidence says that toys symbolically related to healthcare may influence anxiety levels after surgery. The intervention was a specific play intervention for the intervention group (used for two hours after the consciousness recovery) and was not included in the routine care.	After the intervention, pain was measured using the FLACC scale at three moments: after regaining consciousness, one hour after it, and two hours after it. The pain decreased in both groups, but the mean of the experimental group was always lower than that in the control group. The last measure difference was statistically significant.	1b/A
Artilheiro et al., 2011 [23] Brazil	Descriptive, quantitative study	*n* = 30 children undergoing outpatient chemotherapy. Age from 3 to 6 years. 53.3% boys.	Play therapy is applied to children on outpatient chemotherapy treatment. Before medication administration, the child was invited to play with a doll and the materials used in the chemotherapy (IV catheter, cotton, syringe...). The researcher showed the child chemotherapy procedures while telling a story and inviting them to repeat the game. The duration of the session is not specified.	After the intervention, the children showed a positive attitude, cooperating with the procedures and with health staff, establishing a relation of trust with them, presenting a relaxed posture, and smiling during the session.	4/C
Caleffi et al., 2016 [17] Brazil	Descriptive, qualitative study	*n* = 5 children hospitalized. Age: 5 to 8 years.	The play therapy model called “Care with Play” (using toys, dolls representing family and healthcare professionals, hospital supplies, and drawing and painting supplies) was used. It has three stages (welcoming, playing, and concluding) that can be done in one or more sessions. The welcoming is centered in establishing a link with the child and determining the required care. The playing phase has a more direct interaction to determine care deficits, and the concluding phase is when the deficits have been covered and the child is referred to another professional.	Three categories were identified: meanings attributed by children to hospitalization and its influence on nursing care, the perception of therapeutic procedures through therapeutic play, and the importance of the family in care. Play therapy helps the child to change their negatives views about the hospital environment and health professionals. It also decreases fear and helps them to understand the need for hospitalization.	4/C
Dantas et al., 2016 [18] Brazil	Descriptive, qualitative study	*n* = 9 hospitalized children. Age: 4 to 8 years.	An instructional therapeutic play for 20–35 min after intravenous drug administration. A name for the doll was chosen by the child and then a story about the doll becoming sick and needing hospitalization was told to the child. The child was encouraged to assign symptoms and choose what to do with the available material. The child chose simulating venous puncture and intravenous medication administration. This intervention was extra to routine care.	The behavior of the children was observed during the administration of IV medication before the play therapy and 2 to 12 h after. Children aged 4–6 years show more rejection and fear of IV medication administration before applying the game. It was seen that after the intervention, the children were calmer and more showed more confidence.	4/C
Fonseca et al., 2014 [22] Brazil	Descriptive study, qualitative, phenomenology	*n* = 5 children with cancer. Age: 3 to 6 years. 80% girls.	Individual dramatic play therapy sessions were performed and recorded. The play therapy sessions were about a child with cancer using toys (dolls, cars, hospital supplies…)	In the sessions, it was seen that cancer produces feelings of fear, anxiety, and insecurity for the future. The game helped children to express and reduce these emotions.	4/C
Kiche and Almeida, 2009 [13] Brazil	Descriptive, quantitative study	*n* = 34 children who were surgically treated. Mean age: 6 years. 58.51% boys.	A first dressing change was made to the child, observing their reactions. Subsequently, an instructive therapy game was performed, with a professional simulating the dressing change technique in a doll (instructional therapeutic toy) next to the child and inviting them to repeat it. The next day, the simulation was performed again before changing the child’s dressing and observing their reaction. The intervention was done for two months as a routine for children admitted for minor surgeries.	After the intervention, it was observed that during dressing changes, the child was more relaxed, the expression of pain on their face was lesser, and the child smiled and collaborated with the professional. In addition, when measuring pain after surgery, the scores were lower than the previous ones.	4/C
Melo et al., 2010 [24] Brazil	Descriptive, qualitative study	*n* = 7 children with cancer. Age: 5.83 years.	When children attended outpatient chemotherapy, they did individual play sessions in the toy library for 1 to 3 h. The intervention was added to the usual routine.	Playing activities promoted the integral and continuous development of children despite the situation. In addition, they were involved in the disease and collaborated in their treatment.	4/C
Santiago et al., 2015 [19] Brazil	Descriptive, quantitative study	*n* = 21 hospitalized children. Age: 3 to 6 (52.3%) and 7 to 12 (47.3%). 61.9% girls.	The behavior of children during venous puncture was observed. Subsequently, a play therapy session was performed with the children simulating a venous puncture on a doll, and then they repeated the procedure and expressed their doubts. The duration of the play therapy is not specified. The behavior of the children was observed again during an intravenous puncture after applying again the play therapy. The time between both sessions was less than 48 h. Children included in the study were hospitalized for 24 h at least.	The intervention was evaluated observing the children reactions. After the play therapy session, there was an increase in variables that express greater adaptation and acceptance to the puncture process like shouting, muscle tension, or crying.	4/C
Yayan et al., 2019 [11] Turkey	Quasiexperimental study	*n* = 130 children in the postoperative period. Mean age: 7.6 ± 3.48 years. 76.5% boys.	Preparation game: play with dolls and animal figures. Pain relief game: play with dolls, cars, breathing exercises, and massages. Distraction game: singing, puzzles, video games, watching videos, crafting your own toys with clinical material. The intervention was added to the usual care routine.	The play therapy decreased the mean levels of pain in children and anxiety in parents.	2b/B

Note: EL = evidence level; FLACC scale = Face, Legs, Activity, Cry, Consolability scale; IV = intravenous; QT = chemotherapy; RG = recommendation grade.

## References

[1] Junta de Andalucía Carta Europea de los Niños Hospitalizados. https://www.juntadeandalucia.es/organismos/saludyfamilias/areas/sistema-sanitario/derechos-garantias/paginas/carta-nino-hospitalizado.html.

[2] Al-Yateem N.S., Banni Issa W., Rossiter R. (2015). Childhood Stress in Healthcare Settings: Awareness and Suggested Interventions. Issues Compr. Child. Adolesc. Nurs..

[3] Rockembach J.A., Espinosa T., Cecagno D., Thumé E., Soares D. (2017). Inserção do lúdico como facilitador da hospitalização na infância: Percepção dos pais. J. Nurs. Health.

[4] Nicola G., Ilha S., Dias M., Freitas H., Backes D., Gomes G.C. (2014). Perceptions of the caregiver family member about playful care of the hospitalized child. J. Nurs. UFPE.

[5] Marques P., Garcia M., Anders J.C., Homem L., Rocha P., Souza S. (2016). Playful activities in health care for children and adolescents with cancer: The perspectives of the nursing staff. Esc. Anna Nery.

[6] Koukourikos K., Tzeha L., Pantelidou P., Tsaloglidou A. (2015). The Importance of Play During Hospitalization of Children. Mater. SocioMed..

[7] Gesteira E.R., Gonçalves D.S., Marques F., Simões F.D. (2011). Students’ experience for using therapeutic play at practical pediatric nursing. J. Nurs. UFPE.

[8] Seus A.C., Milbrath V.M., Freitag V.L. (2018). Percepción del equipo de enfermería sobre el enfoque lúdico al niño hospitalizado. Cult. Cuid..

[9] Maia E.B.S., Ribeiro C.A., de Borba R.I.H. (2011). Compreendendo a sensibilização do enfermeiro para o uso do brinquedo terapêutico na prática assistencial à criança. Rev. Esc. Enferm..

[10] Gomes I.P., Collet N., dos Reis P.E. (2011). Ambulatório de quimioterapia pediátrica: A experiência no aquário carioca. Texto Context Enferm..

[11] Yayan E.H., Zengin M., Düken M.E., Suna Dağ Y. (2020). Reducing Children’s Pain and Parents’ Anxiety in the Postoperative Period: A Therapeutic Model in Turkish Sample. J. Pediatr. Nurs..

[12] Ullán A.M., Belver M.H., Fernández E., Lorente F., Badía M., Fernández B. (2014). The effect of a program to promote play to reduce children’s post-surgical pain: With plush toys, it hurts less. Pain Manag. Nurs..

[13] Kiche M.T., Almeida F.A. (2009). Brinquedo terapêutico: Estratégia de alívio da dor e tensão durante o curativo cirúrgico em crianças. ACTA Paul. Enferm..

[14] He H.G., Zhu L., Chan S.W., Liam J.L., Li H.C., Ko S., Kalinin P., Wang W. (2015). Therapeutic play intervention on children’s perioperative anxiety, negative emotional manifestation and postoperative pain: A randomized controlled trial. J. Adv. Nurs..

[15] Li W.H., Chan S.S., Wong E.M., Kwok M.C., Lee I.T. (2014). Effect of therapeutic play on pre- and post-operative anxiety and emotional responses in Hong Kong Chinese children: A randomised controlled trial. Hong Kong Med. J..

[16] Teksoz E., Bilgin I., Madzwamuse S.E., Oscakci A.F. (2017). The impact of a creative play intervention on satisfaction with nursing care: A mixed-methods study. J. Spec. Pediatr. Nurs..

[17] Caleffi C.C., Rocha P., Anders J.C., Souza A.I., Burciaga V.B., Serapião L. (2016). Contribution of structured therapeutic play in a nursing care model for hospitalised children. Rev. Gauch. Enferm..

[18] Dantas F.A., Medeiros V., Acioli E., Collet N. (2016). Use of therapeutic play during intravenous drug administration in children: Exploratory study. Online Braz. J. Nurs..

[19] Santiago I.C., de Oliveira J., Bezerra E., Leite K.V., Sousa P.K., Pimentel F.G. (2016). Brinquedo terapêutico no procedimento de punção venosa: Estratégia para reduzir alterações comportamentais. Rev. Cuid..

[20] Al-Yateem N., Rossiter R.C. (2017). Unstructured play for anxiety in pediatric inpatient care. J. Spec. Pediatr. Nurs..

[21] Li W.H., Chung J.O., Ho E.K., Chiu S.Y. (2011). Effectiveness and feasibility of using the computerized interactive virtual space in reducing depressive symptoms of Hong Kong Chinese children hospitalized with cancer. J. Spec. Pediatr. Nurs..

[22] Fonseca M.R.A., Campos C.J.G., Ribeiro C.A., Toledo V.P., Melo L.L. (2015). Revelando o mundo do tratamento oncológico por meio do brinquedo terapêutico dramático. Texto Context Enferm..

[23] Artilheiro A.P.S., De Amorim Almeida F., Chacon J.M.F. (2011). Uso do brinquedo terapêutico no preparo de crianças pré-escolares para quimioterapia ambulatorial. ACTA Paul. Enferm..

[24] Melo L.L., do Valle E.R.M. (2010). A brinquedoteca como possibilidade para desvelar o cotidiano da criança com câncer em tratamento ambulatorial. Rev. Esc. Enferm..

[25] Kool R., Lawver T. (2010). Play Therapy considerations and applications for the practitioner. Psychiatry.

[26] Marques D.K.A., da Silva K.L.B., de Cruz D.S., De Souza I.V.B. (2015). Benfefício da aplicação do brinquedo terapêutico: Visão dos enfermeiros de um Hospital Infantil. Rev. Arq. Ciências Saúde.

[27] Jansen M.F., dos Santos R.M., Favero L. (2010). Benefícios da utilização do brinquedo durante o cuidado de enfermagem prestado a criança hospitalizada. Rev. Gauch. Enferm..

[28] Malaquias T., Baena J.A., Campos A.P., Moreira S.R.K., Bladissera V.D.A., Higarashi I.H. (2014). O uso do brinquedo durante a hospitalização infantil: Saberes e práticas da equipe de enfermagem. Ciência Cuid. Saúde.

[29] Oliveira C., Maia E., Borba R., Ribeiro C. (2015). Brinquedo Terapêutico na assistência à criança: Percepção de enfermeiros das unidades pediátricas de um hospital universitário. Rev. Soc. Bras. Enferm. Ped..

[30] Li W.H.C., Chung J.O.K., Ho K.Y., Kwok B.M.C. (2016). Play interventions to reduce anxiety and negative emotions in hospitalized children. BMC Pediatr..

[31] Depianti J.R.B., Da Silva L.F., Carvalho A.D.S., Monteiro A.C.M. (2014). Nursing perceptions of the benefits of ludicity on care practices for children with cancer: A descriptive study. Online Braz. J. Nurs..

[32] Witt S., Escherich G., Rutkowski S., Kappelhoff G., Frygner-Holm S., Russ S., Bullinger M., Quitmann J. (2019). Exploring the Potential of a Pretend Play Intervention in Young Patients with Leukemia. J. Pediatr. Nurs..

[33] Li H.C., Lopez V. (2008). Effectiveness and appropriateness of therapeutic play intervention in preparing children for surgery: A randomized controlled trial study. J. Spec. Pediatr. Nurs..

[34] Buyuk E.T., Bolişik B. (2015). The Effect of Preoperative Training and Therapeutic Play on Children’s Anxiety, Fear, and Pain. J. Pediatr. Surg. Nurs..

[35] Schleisman A., Mahon E. (2018). Creative play: A nursing intervention for children and adults with cancer. Clin. J. Oncol. Nurs..

[36] Tse M.M.Y., Ng S.S.M., Lee P.H., Lai C., Kwong E., Liu J.Y.W., Yuen J., Bai X., Yeung S.S.Y. (2016). Play Activities Program to Relieve Chronic Pain and Enhance Functional Mobility and Psychological Well-Being for Frail Older Adults: A Pilot Cluster Randomized Controlled Trial. J. Am. Geriatr. Soc..

[37] Saywell N., Taylor N., Rodgers E., Skinner L., Boocock M. (2017). Play-based interventions improve physical function for people with adult-acquired brain injury: A systematic review and meta-analysis of randomised controlled trials. Clin. Rehabil..

[38] Brand S.R., Pickard L., Mack J.W., Berry L.L. (2016). What Adult Cancer Care Can Learn from Pediatrics. J. Oncol. Pract..

[39] Nadler H.S. (1983). Art experience and hospitalized children. Child Health Care.

[40] Ng Q.X., Ho C.Y., Koh S.S., Tan W.C., Chan H.W. (2017). Doll therapy for dementia sufferers: A systematic review. Complement Ther. Clin. Pract..

[41] Braden B.A., Gaspar P.M. (2015). Implementation of a baby doll therapy protocol for people with dementia: Innovative practice. Dementia.

